# Supplementary material for: Prevalence of anxiety and depression during COVID-19 pandemic among healthcare students in Jordan and its effect on their learning process: A national survey
Source: PLoS One. 2021 Apr 5;16(4):e0249716. doi: 10.1371/journal.pone.0249716 (PMC8021185; doi:10.1371/journal.pone.0249716)
Supplement: S1 Appendix — (DOCX) [file pone.0249716.s001.docx]

**Prevalence of anxiety and depression following COVID-19 among healthcare students in Jordan and its effect on their learning process: a national survey**

Dear student,

This cross sectional study involves a short survey, designed by a specialized research team from Applied Science University and the Jordan University of Science and Technology, to assess the prevalence of anxiety/depression following COVID-19 among healthcare students and its effect on their learning.

Please complete the survey, it takes no longer than 5 min. Participation in this study does not pose any risk to participants and is voluntary. Data collected will be strictly confidential and results will only be used for research purposes, aiming to provide evidence based recommendations to policy makers to reduce the occurrence of anxiety/depression among medical students in the future.

**Part 1. Demographics characteristics**

| **Parameter** |
| --- |
| **Age ………………..** |
| **Gender**   - Male - Female |
| **Study Field**   - Medicine - Dentistry - Pharm.D - Pharmacy - Nursing - Other |
| **Level of study**   - Higher education – PhD - Higher education – Masters - Undergraduate education |
| **Nationality**   - Jordanian - No-Jordanian, please state ------------------- |
| **Social Status**   - Single - Married with no children - Married with children |
| **Family Monthly Income level**   - < 500 JD - 500 – 1000 JD - >1000 JD - >2000 JD - >3000 JD |
| **Living place**   - City - Rural - Village - Badiah |
| **Smoking (during COVID_19)**   - None - 1 packet per day - > 1 packet per day |
| **Caffeine intake (during COVID_19)**   - None - < 2 cups - 2-4 cups - > 4 cups |
| **Sleeping hours (during COVID_19)**   - < 4 hr - 4-6 hr - 6-8 hr - >8 hr |
| **Sleeping pattern (during COVID-19)**   - **Mostly during day time** - **Mostly during night time** |

**Part 2. Student’s Anxiety and Depression Assessment**

| **Statement** | **Please choose your answer** |
| --- | --- |
| **Anxiety assessment** |  |
| **I feel tense or wound up** |  |
| Most of the time |  |
| A lot of the time |  |
| From time to time, occasionally |  |
| Not at all |  |
| **I get a sort of frightened feeling as if something awful is about to happen** |  |
| Very definitely and quite badly |  |
| Yes, but not too badly |  |
| A little, but it doesn't worry me |  |
| Not at all |  |
| **Worrying thoughts go through my mind** |  |
| A great deal of the time |  |
| A lot of the time |  |
| From time to time, but not too often |  |
| Only occasionally |  |
| **I can sit at ease and feel relaxed** |  |
| Definitely |  |
| Usually |  |
| Not Often |  |
| Not at all |  |
| **I get a sort of frightened feeling like 'butterflies' in the stomach** |  |
| Not at all |  |
| Occasionally |  |
| Quite Often |  |
| Very Often |  |
| **I feel restless as I have to be on the move** |  |
| Very much indeed |  |
| Quite a lot |  |
| Not very much |  |
| Not at all |  |
| **I get sudden feelings of panic** |  |
| Very often indeed |  |
| Quite often |  |
| Not very often |  |
| Not at all |  |
| **Depression assessment** |  |
| **I still enjoy the things I used to enjoy** |  |
| Definitely as much |  |
| Not quite so much |  |
| Only a little |  |
| Hardly at all |  |
| **I can laugh and see the funny side of things** |  |
| As much as I always could |  |
| Not quite so much now |  |
| Definitely not so much now |  |
| Not at all |  |
| **I feel cheerful** |  |
| Not at all |  |
| Not often |  |
| Sometimes |  |
| Most of the time |  |
| **I feel as if I am slowed down** |  |
| Nearly all the time |  |
| Very often |  |
| Sometimes |  |
| Not at all |  |
| **I have lost interest in my appearance** |  |
| Definitely |  |
| I don't take as much care as I should |  |
| I may not take quite as much care |  |
| I take just as much care as ever |  |
| **I look forward with enjoyment to things** |  |
| As much as I ever did |  |
| Rather less than I used to  Definitely less than I used to |  |
| Hardly at all |  |
| **I can enjoy a good book or radio or TV program** |  |
| Often |  |
| Sometimes |  |
| Not often |  |
| Very seldom |  |

***Scoring: Total score: Depression (D) ___________ Anxiety (A) ______________*

*0-7 = Normal; 8-10 = Borderline abnormal (borderline case) ; 11-21 = Abnormal (case)*

**Part 3. Learning during COVID-19**

| Please choose the best answer that describes your learning during the pandemic (second semester 2019/2020). | | | | | |
| --- | --- | --- | --- | --- | --- |
| **Statements** | **Excellent** | **Very good** | **Good** | **Fair** | **Not good** |
| 1-How do you rank the online learning with regards to theory courses you received last semester compared to last year |  |  |  |  |  |
| 2- How do you rank the online learning with regards to practical courses you received last semester compared to last year |  |  |  |  |  |
| 3-How do you rank the online learning with regards to the assessment and exams you received last semester compared to last year |  |  |  |  |  |
| 4-How do you rank the learning with regards to your training last semester compared to last year |  |  |  |  |  |
| 5-How do you rank the online learning with regards to your relationship with your doctors last semester compared to last year’s |  |  |  |  |  |
| 6-How do you rank the online learning with regards to its effect on your last semester **Grade Point Average** compared to last year’s second semester |  |  |  |  |  |
| 7- What was your **semester** **Grade Point Average** total mark for **LAST** year’s second semester **(2019-2020)**  **­­­­­­­……………………..** | | | | | |
| 8-What was your **semester** **Grade Point Average** total marks for **THIS** year’s second semester **(2020-2021)**  **…………………….** | | | | | |
| 1. **How many hours -in average- you used to study daily BEFORE COVID-19 ?**   **….………………….** | | | | | |
| 1. **How many hours -in average- you used to study daily AFTER COVID-19 ?**   **….………………….** | | | | | |
